# Supplementary material for: Broad Cross-Reactive IgA and IgG against Human Coronaviruses in Milk Induced by COVID-19 Vaccination and Infection
Source: Vaccines (Basel). 2022 Jun 20;10(6):980. doi: 10.3390/vaccines10060980 (PMC9229351; doi:10.3390/vaccines10060980)
Supplement: Supplementary file 1 [file vaccines-10-00980-s001.zip › vaccines-1754991-supplementary.pdf]

Supplementary Material

Table S1. The demography of two cohorts in this study.

		Infection cohort	Vaccinated Cohort
Total N		45	30
Maternal Age (range)		29.9 ± 4.4 (20-38)	33.0 ± 3.4 (25 – 42)
	American Indian	1 (2.1%)	0
Race (%)	Asian	0	1 (3.3%)
	Black	2 (4.2%)	0
	White	40 (85.1%)	29 (96.7%)
	Other	2 (4.2%)	0
	More than One	1 (2.1%)	0
	Prefer not to Answer	1 (2.1%)	0
Ethnicity (%)	Hispanic	12 (25.5%)	0
	Non-Hispanic	24 (51.1%)	26 (86.7%)
	Not reported	11 (23.4%)	4 (13.3%)
BMI (kg/m2) (range)		28.8 ± 6.4 (20.2 – 45.2)	27.1 ± 6.8 (19.9 - 45.1)
Parity		2.2 ± 1.1	2.3 ± 1.3
Birth Weight (g) (range)		3473 ± 439 (2735 - 4621)	3236 ± 540 (1644 - 4046)
Infant sex	Male	24 (51%)	13 (43.3%)
	Female	23 (49%)	17 (56.7%)
Infant age (months) (range)		3.1 ± 2.2 (0.2 – 9.0)	7.5 ± 5.2 (0.5 – 23.4)
Breastfeeding Exclusivity at day0 or post dose 1		0.92 ± 0.18	0.96 ± 0.15
HM feeds (%)		36/46 (78%)	27/30 (90%)
Vaccine	BNT162b2 (Pfizer-BioNTech)		12 (40%)
	mRNA-1273 (Moderna)		18 (60%)
Household Member Diagnosed COVID-19		28/47 (59.6%)	5/30 (16.7%)
Infant Ever Tested		17 (36%)	13/30 (43%)
Infant Ever Diagnosed		9 (53% tested, 19% total)	2/13 (15% tested, 6.7% total)

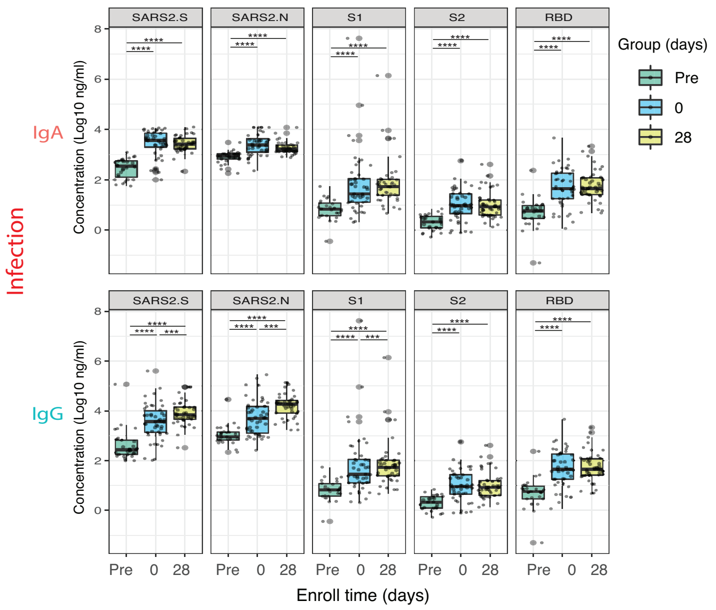

Figure S1. Supplementary figure 1: Anti-SARS-CoV-2 IgG serum antibody response after SARS-CoV-2 infection. In the infection cohort, the IgG antibodies in fingerstick blood samples had evaluated by mPLEX-CoV assay. All samples were tested at the same time in duplicate, and significant results were calculated with generalized linear mixed-effects models (**** P < 0.0001, *** P< 0.001, ** P<0.01, * P<0.05).

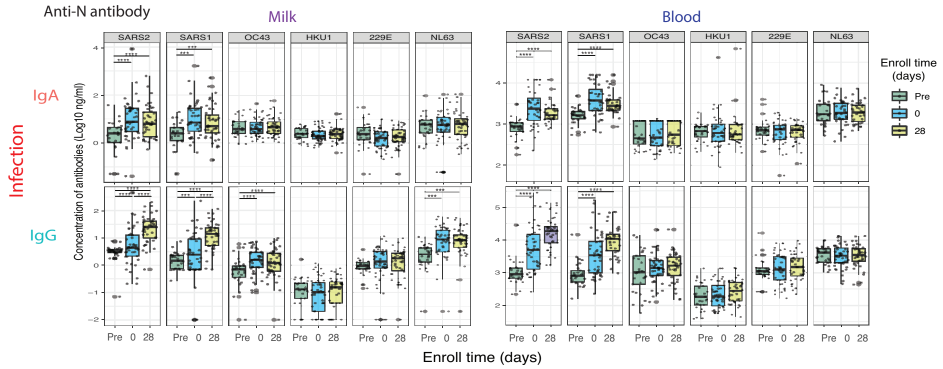

Figure S2. Supplementary figure 2: Anti-N HCOV antibody response in human milk and blood after SARS-CoV-2 infection. In the infection cohort, the anti-N IgG antibodies against SARS2, SARS1, and other common coronaviruses (HCoVs) in the milk and fingerstick blood samples had evaluated by mPLEX-CoV assay. All samples were tested at the same time in duplicate, and significant results were calculated with generalized linear mixed-effects models (**** P < 0.0001, *** P< 0.001, ** P<0.01, * P<0.05).

Table S2. The mPlex-CoV assay panel of human coronaviruses.

Type	Coronavirus	Protein	Abbreviation	textbfGene Acc
β	SARS-CoV-2	S	SARS2	EPI_ISL_402124
	SARS-CoV-1	Protein	SARS1	NC_004718.3
	HCoV-OC43		OC43	KX344031.1
	HCoV-HKU1		HKU1	AY597011
α	HCoV-229E		229E	KY369911.1
	HCoV-NL63		NL63	NC_005831.2
β	SARS-CoV-2	N	SARS2	EPI_ISL_402124
	SARS-CoV-1	Protein	SARS1	NC_004718.3
	HCoV-OC43		OC43	KX344031.1
	HCoV-HKU1		HKU1	AY597011
α	HCoV-229E		229E	KY369911.1
	HCoV-NL63		NL63	NC_005831.2

[a] Spike (S) proteins of human coronaviruses are recombinant proteins expression by the baculovirus system with the trimerization domain at the C-terminal with His-tag.

[b] Nucleocapsid proteins of human coronaviruses are recombinant proteins with C-terminal His-tag .
